# Supply—Demand

**Published:** 1879-11

**Authors:** 


*Supply—Demand.*—The Editor of the New York *Medical Record* sharply says in an article on "the overcrowded profession" that "where there are a few Medical Colleges in a State, they supply a demand; where there are many, they demand a supply; get it by superior inducements in the line of economy to the student of time and money." and Prof. Alfred Mercer, in an interesting address recently made to the council of the Syracuse (N. Y.) University, very pertinently says:

"From the cheapness of American diplomas, and from the few unenforced legal restrictions on the practice of medicine, with or without a diploma, or any known qualifications whatever, we have one doctor to every six hundred inhabitants, while a few miles from here, just over the Canadian border, they have only one to 1,200 inhabitants, while in Great Britain there is but one to 1,672.

| | | |
|---|---|---|
| France has one to | - - - - - | 1,814 |
| Germany " " | - - - - - - | 3,000 |
| Belgium " " | - - - - - | 2,048 |
| Austria " " | - - - - - - | 2,500 |
| Italy " " | - - - - - | 3,500 |
| Norway " " | - - - - - - | 3,480 |

Thus, we have two doctors in the United States to one in Canada, nearly three to one in Great Britain, more than four to one in France, and five to one in Germany. The just relative proportion of doctors to population has been variously estimated at from fifteen to twenty-five hundred. The present average of the civilized world would probably fall within these limits."

It is perhaps a nice question to decide *when* a profession is crowded, but that that of medicine is getting so very rapidly is obvious; and in proportion as the wedging in process goes on, and the question becomes one of daily bread; practitioners may not hope for any courteous treatment from each other. Dentistry may well take warning from medicine—dentistry which is more exposed to attack from the more nearly mechanical nature of most of its work, take warning and reflect on the tendencies of the times. It is probable, nay, certain, that of the most of those practising in the large cities, not greater pecuniary success is achieved in dentistry than in most of the callings in life; the masses eke out a a precarious livelihood, perish and leave no mark or name. Is this on account of inherent faults in the system by which these practitioners are put into the field of competition with those already struggling with difficulties, or is it that there are always too many seeking a livelihood in this direction? or is it that in spite of the potent fact that teeth are perishing by thousands for want of good dentistry, or saving methods; the public are unawakened to the importance of this, and of the usefulness of those who are seeking practice.

Hard questions, all of them, and the solution of the first one alone is perhaps difficult. The systems of teaching are faultly. Partly so on account of want of outside pressure, forcing up the standard, for the Schools are mainly what the profession at large make them, and partly on account of the fact that those teaching are not made independent of the classes in point of pay. Competition of Schools, the pressure from those representing applicants for matriculation—practitioners, etc;—the sad want of preliminary training on the part of matriculants themselves; the fact that many who desire to enter also desire work at once on account of poverty and so cut themselves off from careful theatrical training—all these are facts which tend to lower the standard of graduation, and exert a pressure, which no faculty, unless unhampered by liberal endowments, can ignore or avail. No man with eyes open can fail to see how in our large cities the best teaching is done in the public schools, where teachers are independent of patronage; and the law is a general one, and will have its effect so long as human nature is what it is.

